# The decidua—the maternal bed embracing the embryo—maintains the pregnancy

**DOI:** 10.1007/s00281-016-0574-0

**Published:** 2016-06-10

**Authors:** Mayumi Mori, Agnes Bogdan, Timea Balassa, Timea Csabai, Júlia Szekeres-Bartho

**Affiliations:** 1 Department of Obstetrics and Prenatal Medicine, University Medical Center Hamburg-Eppendorf, Martinistr. 52, 20246 Hamburg, Germany; 2 Department of Medical Biology, Medical School, Pécs University, János Szentágothai Research Centre, University of Pécs, Szigeti Str. 12, H-7643 Pécs, Hungary; 3 MTA - PTE Human Reproduction Research Group, Pécs, Hungary

**Keywords:** Decidua, Endometrium, Pregnancy, Infertility, Preeclampsia, Vascular remodeling

## Abstract

The decidua has been known as maternal uterine tissue, which plays essential roles in protecting the embryo from being attacked by maternal immune cells and provides nutritional support for the developing embryo prior to placenta formation. However, there are questions that still remain to be answered: (1) How does the decidua supply nutrition and provide a physical scaffold for the growing embryo, before placental vascular connection is established? (2) How is the balance between preventing an anti-embryo immune response and protecting both embryo and mother from infections established? To understand basic personas in decidual tissues, we review the structure of the decidua composed of terminally differentiated uterine stromal cells, blood vessels, and a number of repertoire of uterine local immune cells, including the well-known uterine natural killer (uNK) cells and recently discovered innate lymphoid cells (ILCs). Decidual macrophages and uterine dendritic cells (DCs) are supposed to modulate adaptive immunity via balancing cytokines and promoting generation of regulatory T (T_reg_) cells. During decidualization, vascular and tissue remodeling in the uterus provide nutritional and physical support for the developing embryo. Secretion of various cytokines and chemokines from both the embryo and the decidual cells activates multiple signaling network between the mother and the embryo upon implantation. Defects in the decidual development during early pregnancy result in loss of pregnancy or complications in later gestational stage.

## Introduction

The decidua is a transient but important platform in the uterine tissue, which comprises terminally differentiated endometrial stromal cells, newly generated maternal vascular cells, and maternal blood cells inside and outside the vessels. Development of the decidua after attachment of the blastocyst on uterine wall is a drastic tissue remodeling, involving physical and humoral changes in the residential and recruited immune cells. Indispensability of the decidual tissue for establishing implantation of the embryo and maintaining pregnancy until the stage of placenta formation was first indicated by its physical importance when mice were challenged by blastocyst transfer into the peritoneal cavity and failed [1], and when tubal decidual formation was observed in some cases of human ectopic implantation in the ovary or peritoneal cavity [2], and when embryonic loss was observed by ovariectomy-mediated progesterone withdrawal due to “collapse” of the rat decidua [3]. Thus, decidualization of uterine tissue is essential to establish successful pregnancy, but how is it generated, and how does it affect embryonic growth?

**Fig. 1:**
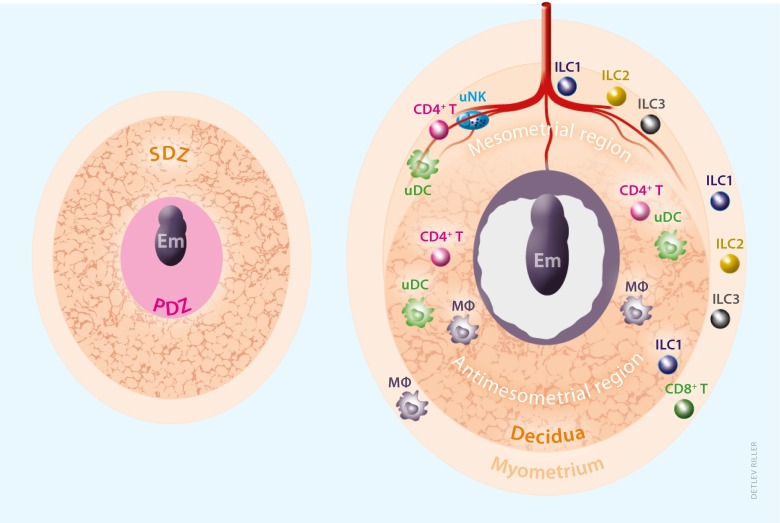
Anatomical localization of maternal immune cells in murine decidua. Due to the difficulty in obtaining specimens from normal pregnant women at the earliest stage of pregnancy, tissue distribution of maternal immune cells in murine uterus at gestational day (gd) 5.5 and 7.5 is shown. The primary decidual zone (PDZ) is avascular and CD45^+^ cells are scarcely found [4]. Secondary decidual zone (SDZ) at gd 5.5 and antimesometrial decidua at gd 7.5 are rich in small blood vessels, whereas mesometrial region at gd 7.5 is surrounded by lateral dilated large vessels (not shown). In the mesometrial decidua, uNK cells are most abundantly found [5], also see Fig. 2], DCs are confined in the entire decidua [6]. In addition to uILC1 in the decidua, other uILCs are detected in mesometrial region and myometrium [7], but the interaction of uILCs with vasculature or other immune cells have not been reported. Except for the report on suppressed infiltration of cytotoxic effector T cells in the decidua [8], T cell subsets inside the murine decidua have not been well known. Macrophages can be found in the region between trophoblast and uterine stromal cells in association with vascular endothelial cells [4]. Note that the numbers of representative cell types are not consistent with the actual populations

The decidual parenchymal cells, hereinafter called as decidual cells, are derived from uterine stromal fibroblast-like cells in the endometrium. They are large, round, and multi-nuclear polyploid cells, rich in glycogen and lipids, and produce a variety of functional markers such as prolactin and its related family proteins, and insulin-like growth factor binding proteins (IGFBPs). The mouse embryo at blastocyst stage attaches to the uterine lumen on gestational day (gd) 4.0 post-coitum, and the primary decidual zone (PDZ) is immediately established at the endometrial layer closest to the implanted embryo (Fig. 1). This is considered to be the first protective scaffold for the embryonic growth with avascular and tight cellular composition [9]. By gd 5.5, PDZ is completed, while the secondary decidual zone (SDZ), surrounding the embryo and PDZ, is developing into terminal differentiation of the decidual stromal cells, starting from antimesometrial toward mesometrial region. In contrast, mesometrial region seen from gd 6.5 consists of highly dilated large vasculature, and both mesometrial and antimesometrial decidual regions decrease from gd 8.5 along with placenta formation [10]. Concomitant angiogenesis in the decidualizing endometrium and spiral artery remodeling during early pregnancy both in human and rodents strongly suggests that nutrition supplied by maternal blood vessels is essential for early embryonic growth before placental connection.

## Development of the decidua and its molecular mechanisms

### Regulators for decidualization outside and inside uterine stroma

Cyclic fluctuation of endometrial cellular differentiation and subsequent apoptotic death in human uterus are under hormonal control. In the absence of entopic embryo in the uterus, human decidua can be formed routinely and then shed off (like leaves on a *deciduous* tree, which is the origin of the word “decidua”). In rodents, artificial decidualization can be observed as deciduoma reaction after a mild physical stimulation of pseudo-pregnant uterus without embryos. Compared to the pregnant uterus, various deciduoma models in mice show more expanded sizes of the uterus and slight differences in expression of decidual markers such as alkaline phosphatase 2 and Wnt4 [11]. Signaling between the embryo and uterine luminal epithelia, mediated by adhesion molecules such as integrins and carbohydrate moieties on glycoproteins, leading to luminal epithelial apoptosis and subsequent interaction between the epithelium and the proximal stromal cells, is also considered to be engaged in initial decidual differentiation [12].

By use of artificial deciduoma in rodents stated above, impaired decidualization has been demonstrated in mice deficient of progesterone receptors (PR) or PR-related pathways (Bmp2, Wnt4, Hoxa10, and Hoxa11) [13–16], indicating that the endocrine system plays the major role in decidualization [17]. Other pathways involved in the acceptance of blastocysts by local and systemic regulation in the maternal uterine epithelium and endometrium are reviewed elsewhere [18]. A mouse model deficient in interleukin-11 (IL-11) cytokine signaling (IL-11Rα^−/−^) [19] shows impaired decidualization, accompanied by reduced uterine stromal cellular proliferation [20]. Once proliferation and differentiation of decidual cells are initiated, they proceed to multi-nuclearization, i.e., DNA replication without cell division (endo-reduplication/polyploidy), which allows expression of multiple genes and secretion of the translated proteins with less energy consumption, and is considered to be an important hallmark of decidual maturation in rodents and humans [21, 22]. In the mouse model deficient in Death effector domain-containing protein (Dedd) [23], bidirectional pathways of Akt signaling and cyclin D3/Cdk4/Cdk6 have been shown to contribute to decidual cellular multi-nuclearization. In the absence of Dedd, protein stability of Akt [23, 24] and Cyclin D3/Cdk4/Cdk6 complexes are reduced, corresponding to lower ratio of multi-nuclearization in decidual cells. As demonstrated in human decidual and endometrial cell cultures [25], Akt signaling is associated with decidual differentiation. Das SK et al. has suggested that cyclin D3, in association with Cdk4, Cdk6, and p21, is an essential cell cycle regulator in the endo-reduplication of multi-nuclearizing murine decidual cells [26]. The expression of cyclin D3 and p21 are also indicated to be downstream of IL-11 signaling [27]. Intriguingly, while Dedd^−/−^ female mice cannot produce any progeny, and while IL-11Rα^−/−^ female mice are severely infertile, any single knockout of either Akt1, Akt2, Akt3, or cyclin D3 does not cause complete infertility, suggesting the importance of Dedd as a master regulator in the upstream of these multiple proteins’ network. The connection between endocrine system and cytokine signaling or Dedd pathway has not been clarified.

### Uterine angiogenesis and tissue remodeling

1. Vascular endothelial remodeling modulated via steroid hormones
  In menstrual cycles and before implantation occurs, ovarian steroid hormones, 17β-estradiol (E_2_) and progesterone (P_4_), modulate the uterine vascular development and functions, resulting in drastic changes of volume, elasticity, and nutrient transportation of the entire uterus. E_2_ has more effects on vascular permeability via suppression of adhesion molecules such as E-selectin, vascular cell adhesion molecule-1 (VCAM-1), and intercellular adhesion molecule-1 (ICAM-1) in human umbilical vein endothelial cells (HUVEC) [28]. On the other hand, P_4_ has more angiogenic effects on HUVEC via inducing proliferative factors from endometrial cells, such as vascular endothelial growth factor A (VEGFA), angiopoietin-2 (ANGPT2), and fibroblast growth factor 2 (FGF2) [29]. P_4_ may also directly suppress ICAM-1 expression in HUVEC [30]; however, this effect seems to be minimal in vivo. VEGF is also expressed in endometrial stroma at the secretory phase and in pregnant decidual cells, both in mice and in humans [31, 32]. Angpt2 and other angiogenic factors are also shown to be regulated under intrauterine E_2_ [33]. Rapid angiogenesis itself may cause loosened structure of vascular network, leading to local hyper-permeability, e.g., in a tumor lesion [34]. The action of endocrine factors on vascular endothelial and smooth muscle cells may affect vascular stability to modify exchange of substances such as free fatty acids loaded on plasma albumin and blood cells between blood and stromal tissue, and regulate expression of surface molecules to recruit uterine-specific immune cells.
2. Morphological dynamism in the uterine tissue
  The decidual cells, not only secrete growth factors but also release tissue inhibitor of matrix metalloproteinases (TIMPs) to suppress trophoblast-derived matrix metalloproteinases (MMPs) [35, 36] and express contact-dependent signaling molecules such as connexin 43 (Cx43) [37]. Although not all of the matrix proteins have been shown to be specifically expressed in either the human or murine decidual cells, α_2_-macroglobulin, one of potent MMP inhibitors, has been shown as downstream of IL-11-invoked JAK-STAT3 pathway in rodents [38], which is also implicated in human endometrial stromal cells (hESCs) [39]. A murine model using conditional knockout of Cx43 via PR-Cre recombination showed the importance of uterine stromal Cx43 on vascular endothelial proliferation at gd 7.5 [37]. The uterus deficient in Cx43 shows insufficient deciduoma response accompanied by reduced uterine vascular angiogenesis. This effect is possibly related to the gap-junction communication between decidual cells which secretes VEGF and angiopoietins, but the communication between decidua and vascular endothelial cells remains to be investigated.

## Classical immunology in the decidua

To challenge the immune privileged feature of the decidua against embryonic graft, the first experimental transplantation of a skin allograft was tested on rodent decidua [40]. The allograft survived longer, however, in the end, the graft was rejected in the pre-immunized pregnant rodent. In contrast to another failure in transplantation of paternal skin allograft tested in the rat choriodecidual junction [41], embryos surrounded by putatively paternal antigen-positive trophoblasts are able to be accepted. Early studies also investigated the existence of immunosuppressive substances from murine decidual culture in vitro [42] and of hormone-dependent suppressor cells regardless of implanted embryos [43]. However, these studies could not identify the cell subset or molecules derived from the decidua.

In the modern era, with the development of technologies in flow cytometry and imaging analysis, a number of studies have described the presence, distribution, and the functions of maternal immune cells in the decidual tissue in the early phase of gestation (Fig. 1, Table 1). The decidua contains a large number of maternal immune cells, which supposedly establish the balance between defense against pathogens and a tolerance of the embryo. The major populations include innate immune cells, i.e., uNK cells and macrophages. However, small populations of ILCs and adaptive immune cells cannot be ignored. Moreover, it is important for the future maternal and fetal health that the decidua holding the embryo can provide protective responses against pathogens, without an excessive inflammation or that would harm the embryo/fetus. Looking at the basic functions and characteristics of these immune cells, tracking the outcomes of deficiency or abnormality in each component of these immune subsets, we may be able to shed light on their roles and importance in normal and pathological conditions.

**Table 1:** Composition of uterine immune cells at post-implantation stage in mice

	Virgin	gd 5.5	gd 6.5	gd 7.5∼9.5
uNK	DBA^−^ 10^a^∼20 %^b^	DBA^−^ <5 %^b^	DBA^−^ 8 %^a^	DBA^−^ 8 %^a^
	DBA^+^ 0 %^f^	DBA^+^ N.D.	DBA^+^ <2 %^a, f^	DBA^+^ 20 %^a^
Mϕ	8 %^b^	20 %^b^	30 % ^f^	N.D.
DC	2∼3 %^b,d^	5∼6 %^d^	15 %^f^	15 %^e^
T	<1 % ^f^	N.D.	<1 % ^f^	N.D.
uILCs	Very few ILCs^c^ uILC1 is dominant	N.D.	N.D.	Very few ILCs^c^ uILC3 is dominant

Uterine cellular composition among CD45^+^ leukocytes assessed by flow cytometry are shown. During post-implantation stage of murine pregnancy (gestational day (gd) 5.5∼9.5), the ratios of different subsets change in comparison to virgin uterus. uNK: CD3^−^CD122^+^ [44] or CD49b^+^CD11b^−^ [45] cells. Mϕ: CD11b^+^F4/80^+^ monocyte-derived cells. DC: CD11c^+^ cells [45]. T: CD3^+^CD4^+^ and CD3^+^CD8^+^ T cells. ILCs: defined as in the text [46]. There are no DBA^+^ cells in non-pregnant uterus and low number of DBA^+^ cells at gd 6.5 [44], in contrast to increased DBA^+^ from gd 7.5 [44]

*N.D.* no data is available

^a^Reference [48]

^b^Reference [55]

^c^Reference [85]

^d^Reference [117]

^e^Reference [47]

^f^Source: Mori M et al., unpublished data

### Uterine NK cells

**Fig. 2:**
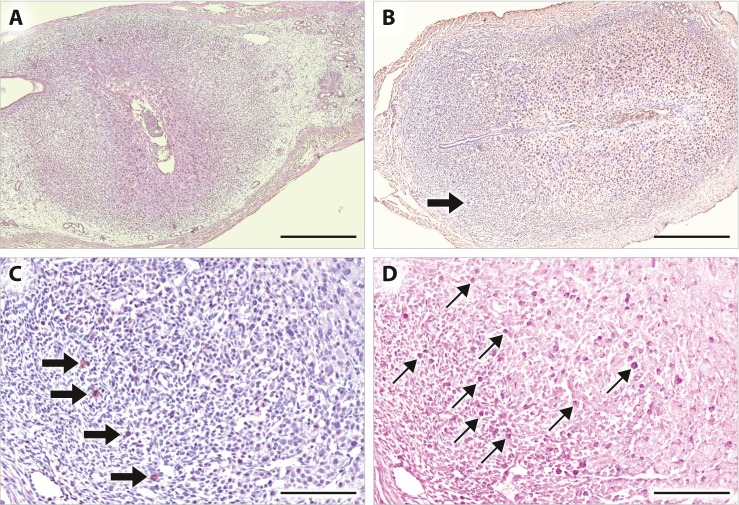
Distribution of uNK cells at early gestational stage in mice. Uterine sections from C57BL/6 female mice at gd 5.5 (**a**) and gd 6.5 (**b**−**d**) were stained with DBA lectin (A−C) or PAS (D). **a** At gd 5.5, DBA^+^ NK cells are scarcely detected. **b** At gd 6.5, DBA^+^ NK cells are increasingly detected in the mesometrial region. **c** Higher magnification of **b. d** Higher number of PAS^+^ NK cells are detected in the continuous section of **c**. *Thin arrows*: PAS^+^ cells, *thick arrows*: DBA^+^ cells. *Bars*, **a**, **b** 500 μm, **c**, **d** 100 μm

Natural killer (NK) cells are derived from pluripotent hematopoietic stem cells in the bone marrow and develop as lymphoid but without receptor gene rearrangement like in the case of T cells. NK cells mediate innate cellular immunity against pathogens and cancer cells. Mature NK cells possess both activating and inhibitory receptors for class I MHC such as Ly49 subtypes in mice and killer inhibitory receptors (KIRs) in human [48]. They also have MHC-independent natural cytotoxicity receptors (NCRs). In humans, peripheral and uterine NK cells represent two phenotypically distinct populations (Table 2). The majority of human peripheral NK cells express low density of CD56 (CD56^dim^) and high levels of the FCγRIII (CD16^+^) indicating ADCC-mediated cytotoxic functions, while the rest of them express high density of the CD56 (CD56^bright^) and are CD16-negative corresponding to low cytotoxicity. uNK cells constitute 60 to 70 % of all decidual lymphocytes in the first trimester of human pregnancy [49]. In contrast to peripheral NK subsets, most decidual NK cells are CD16^−^CD56^bright^. Further to the phenotypic differences, uterine and peripheral NK cells exert different functions. Peripheral CD16^+^CD56^dim^ NK cells are granular and cytotoxic, while the minor peripheral NK subset does not contain cytoplasmic granules and is not endowed with a cytotoxic potential but displays an immuno-regulatory role via cytokine production [50]. Decidual NK cells contain cytotoxic granules [51] and selectively overexpress genes of secreted proteins with known immunosuppressive activity [49], as well as perforin and granzymes A and B [52]. In mice, two distinct uNK cell subsets are distinguished by periodic acid-Schiff (PAS) and Dolichos biflorus agglutinin (DBA) reactivity. PAS^+^DBA^−^ cells produce IFN-γ, which is implicated in maternal spiral arterial remodeling [53], whereas the PAS^+^DBA^+^ population produces angiogenic factors [54–56]. Yadi et al. [44] describe two distinct subsets of CD3^−^CD122^+^ NK cells in the mid-gestational mouse uterus: a small subset similar to peripheral NK cells, and a larger DBA^+^ subset that expresses activating receptor NKp46 and inhibitory receptor Ly49s, but not NK1.1 or DX5. It must be mentioned that via maternal blood which perfuses the placenta, both subsets of peripheral blood NK cells could get in contact with fetal tissues. Uterine NK cells have a low spontaneous cytotoxicity, which is in line with the concerted presence of non-classical MHC molecules on the trophoblast and expression of inhibitory receptors on the NK cells, resulting in decreased degranulation [57]. However, in the presence of pathogens, the proportions of activating and inhibitory receptors may shift to promote cytotoxicity [58]. At present, there is no evidence that the cytotoxic potential of NK cells has any direct effect on trophoblast, while maternal MHC but not paternal MHC has been shown to educate uNK cells via matching Ly49 receptors to produce IFN-γ as a vascular-remodeling factor [59]. Murine uNK cell precursors either originate from outside the uterus [60] or differentiate from resident hematopoietic precursors [61] in the presence of other immune cells. In SCID mice, uNK cell differentiation in the decidua basalis was shown to be delayed during the early placentation period due to a lack of functional T and B cells [62]. The number of the resident NK cell population declines between gd 0.5 and 5.5 [45] and is considered to be replaced by recruited extra-uterine precursors which differentiate into uNK cells. DBA^+^ NK cells are scarce in the decidua at gd 5.5, while from gd 6.5 an increasing number of DBA^+^ and PAS^+^DBA^−^ NK cells can be detected ([63], Fig. 2).

**Table 2:** The major differences between human peripheral NK cells and uNK cells

Peripheral NK cells		Uterine NK cells
(90 %)	(10 %)	
CD56^dim^	CD56^bright^	CD56^bright^
CD16^+^	CD16^−^	CD16^−^
Cytotoxic	Non-cytotoxic	Low cytotoxicity
Granular	Non-granular, cytokine producing	Granular
LFA1^+^		CCR7^+^
Perforin^+^ granzyme^+^		Perforin^+^ granzyme^+^

Interleukin-15 (IL-15) is a critical regulator of NK and uNK cell differentiation [64, 65]. IL-15^−/−^ females lack uNK cells, as well as spiral artery remodeling [66, 67]. A recent study [68], however, compared the gene expression profiles in implantation sites of the uterus during decidualization on gd 7.5 between wild-type and uNK cell-deficient (IL-15^−/−^) mice, and found no different expression of genes involved in decidualization or angiogenesis, with the exception of Adamts9, which is an anti-angiogenic factor, expressed at a higher rate in IL-15^−/−^ than in wild-type mice. Immune cell-deficient murine models showed the significance of NK cell contribution to implantation and normal pregnancy. A murine alymphoid model, Rag2^−/−^Δγc, which lack T, B, and NK cells systemically, have deficient early decidual angiogenesis, delayed early embryonic development, and failure of spiral arterial modification at mid-gestation. All these anomalies are corrected by reconstitution with NK^+^T^−^B^−^ grafts before mating [69]. Effects of cellular contact of uNK cell with other lymphocytes or other decidual-resident cells are largely unknown, but soluble factors from uNK cells such as IFN-γ has been shown to contribute for spiral artery remodeling [70] in NK-deficient mouse models. In humans, uNK cells also generate an array of angiogenic growth factors, e.g., VEGF, ANGPT-1, 2, transforming growth factor-β (TGF-β) [71], and placental growth factor (PlGF) [72, 73]. As a consequence of inadequate spiral artery remodeling, preeclampsia and intrauterine growth restriction could be expected; however, data from different laboratories are conflicting. This is probably due to differences in the focus on peripheral and decidual NK cells and their subsets, and to diversity of effects derived after preeclampsia and abortion, which may trigger cytotoxic NK activation. A recent report has shown that pregnant women with high Doppler resistance index, indicating impaired spiral artery remodeling and higher risk of preeclampsia, possess non-cytotoxic decidual NK cells as well as normal pregnant women, but with decreased expression of KIR2DL/S1, 3, 5, receptors against canonical class I histocompatibility locus antigen C (HLA-C), and LILRB1 against HLA-G on trophoblast [74]. Controversially, in vivo depletion of NK cells by anti-asialo-ganglio-N-tetraosylceramide (asialo-GM1) antibody reduced abortion rates in the abortion-prone CBA/J female mice mated with DBA/2J male mice [75]. This is considered to be due to the double-face of cytotoxic and vascular-remodeling NK cells, for similar NK depletion of CB17 SCID mice lacking effector T and B cells showed increased abortion rate [76]. This is probably due to lack of innate immunity against indigenous bacteria. Thus, when NK cell are depleted systemically, different cytotoxicity in two subsets in uNK cell should be taken into account.

Herein, cytotoxic NK cells might play a role in pregnancy pathologies. The increased resorption rates of pregnant BALB/c mice induced by anti- progesterone-induced blocking factor (PIBF) antibody were corrected by treating the mice with anti NK-1.1 antibody [77]. In humans, increased number of CD56^+^ cells were demonstrated in mid-luteal phase endometrial biopsies from patients with idiopathic recurrent miscarriage (RM) [78] but another study (Tuckerman et al., [79]) concluded that numbers of uNK cells in RM do not predict subsequent pregnancy outcome. Among decidual lymphocytes from failed pregnancies, there were less perforin-positive CD56^+^ cells than in deciduas from normal pregnancies [80], suggesting an increased rate of degranulation taking place in the former cases. In women with RM, a lowered uterine artery resistance to blood flow was demonstrated by Doppler ultrasonography together with an increased percentage of uNK cells during the mid-secretory phase, suggesting a correlation between excessive blood vessel development and the pregnancy failure [81]. Other studies have shown that not the number of endometrial total NK cells but the dominance of CD16^−^CD56^bright^ uNK cell subset was significantly decreased in favor of CD16^+^CD56^dim^ uNK cells in recurrent aborters [82]. Patients who miscarried chromosomally normal embryos had decreased percentage of CD16^−^CD56^bright^ uNK cells compared with those of normal pregnancy [83, 84]. These data suggest that a part of RM with unknown etiology might be explained by deficiency in CD16^−^CD56^bright^ uNK cells, or alternatively by excess infiltration of CD16^+^CD56^dim^ NK cells derived from perfusing blood [85]. However, the causal association and precise mechanism have been unknown especially in humans. Taken all these together, further investigation on functions of uNK cells, rather than number or surface markers, is required.

### Innate lymphoid cells

Innate lymphoid cells (ILCs) play a role in protection against pathogens, in lymphoid organogenesis, and in tissue remodeling. They are now divided to three subsets on the basis of their phenotypic and functional characteristics [86–88]. Group 1 ILCs can be distinguished from cytotoxic NK cells by lack of the transcription factor Eomes, yet they produce IFN-γ via T-bet transcription factor, rendering ILC1s weakly cytotoxic. Group 2 ILCs express a discriminative IL-33 receptor and also express chemokine receptors CCR4 and CCR5 upon stimulation. In response to IL-25, IL-33, parasites, or tissue injury, they produce Th2 cytokines such as IL-4, IL-5, and IL-13, acting in a regulatory fashion similar to Th2 cells. Group 3 ILCs require the transcription factor retinoic acid-related orphan receptor γt (RORγt) for their generation. They produce IL-22 and also secrete IL-17 in certain circumstances. Though they are non-cytotoxic, a subset of ILC3s expresses an NK activating receptor NCR (NKp46/44). All ILCs share a common precursor expressing the ID2 transcription factor. ILCs play a role in innate defenses against pathogens and in lymphoid tissue organization during fetal life [89], but their most important role is to behave as an intermediary between innate immune responses and T helper functions. Expression of both NK receptors and production of Th1, Th2, Th17, and Th22 cytokines by ILCs suggest that they might play a role in establishing the balance between immunity and tolerance both in innate and adaptive settings (Table 3). During pregnancy, the most important role has been attributed to uNK cells as described in the section above. In addition to this, IL-22-producing non-NK ILCs are also present in the non-pregnant uterine mucosa as well as in the decidua during the second trimester [91]. Recently Doisne et al. have identified uterine ILC subsets (uILCs) in human endometrium in the first trimester and in murine uterus at the beginning of placenta formation [7]. Three subpopulations, uILC2, uILC3, and uterine-specific CD127^−^ uILC1 were found in the murine uterus at gd 9.5, but with different distribution within the implantation site (Fig. 1 and [7]). Both in mouse uterus and in human endometrium, uILC3 seems to be the dominant subset during pregnancy, whereas uILC2 is scarcely detected, and decidual uILC1 consists of CD127^−^ cells similar to intraepithelial lymphocytes (IEL) in the intestine [7] (Table 3).

**Table 3:** Comparison of uterine ILC subsets to Th cells and uNK cells

uILC subsets	Comparable Th subsets	Comparable uNK subsets
uILC1 (T-bet, (IFN-γ))	Th1	IFN-γ (DBA^−^ uNK in mice)
uILC2 (IL-33R, IL-7Rα, GATA3, IL-5)	Th2	
uILC3 (RORγt, IL-7Rα, IL-17, 22, partially NCR^+^CD56^+^ in human)	Th17, Th22	Some DBA^+^IL22^+^ in mice? NCR^+^CD16^−^CD56^bright^ in human?

To understand the complicated groups of uILCs, subsets are aligned with regard to comparable functions of helper T cells and uNK cell cells. uILC1s produce IFN-γ, but at a lower level compared to that of uNK cells. Unlike ILC2s in other tissues, uILC2s constitutively express IL-5 [89]. uILC3s partially express IL-17 and IL-22 like Th17 or Th22 cells. Interestingly, some DBA^+^ NK cells are reported to secrete IL-22 [5]. uILC3s in human possess NCRs in addition to CD56 expression, similar to the supported presence of NCR^+^ NK cells within CD16^−^CD56^bright^ population [90]

Due to their relatively recent discovery, the role for these cells in reproduction is yet to be established. Mouse models lacking particular subsets of ILCs, without abrogated Th2 cell differentiation seen in ST2- (IL-33R) deficient mice [46], e.g., irradiated wild-type mice of bone marrow chimera with *staggerer* mutant (RORα sg/sg) mice, are considered as an appropriate model to assess the intrinsic roles of ILC2. RORγt-reporter mice in combination with RAG2^−/−^ genotype are useful to elucidate the roles for ILC3 [92]. Considering the decidua-specific distribution of uILC1s (Fig. 1), PLZF-deficient (lacking all the ILC subsets) mice is also an alternative optional model to investigate the roles of uILCs in the decidua. However, none of these models have been tested for quantitative reproductive capacity, although there has been no report of breeding problems. Further investigation using the ILC-deficient mouse models is demanded to elucidate the specific roles for ILCs in reproduction.

### Decidual macrophages

When pregnancy is established, circulating monocytes infiltrate the decidua and develop into macrophages [93], together with uterine resident myeloid cells, constituting 20 to 25 % of human decidual leukocytes [94]. Mice lacking decidual macrophages are not available. Even CSF-I-deficient osteopetronic (op/op) mice show a small number of F4/80-positive cells in the decidua, although seen only at gd 7.5 [95]. However, op/op female mice crossed with op/op males are infertile at implantation stage and any other combination involving op/+ females or op/+ males result in mild subfertility [96], suggesting that a sufficient number of macrophages is necessary to sustain the pregnancy. In op/op females mated with op/+ males, implantation rate was decreased to 60 % with lower number of implantation sites and lower survival rate of implants until term pregnancy, resulting in 39 % fertility in comparison to op/+ females mated with op/+ or op/op males, showing > 92 % implantation rate and > 85 % fertility.

Based on differential expression of the complement receptor CD11c, two distinct subpopulations (CD11c^hi^ and CD11c^lo^) have been identified in human decidua [96]. In the first-trimester decidua, the majority of the macrophages are CD11c^lo^. These express genes associated with extracellular matrix formation, muscle regulation, and tissue growth [97]. CD11c^hi^ macrophages, constituting approximately one third of the decidual population, express genes associated with antigen-presenting function, e.g., CD1a, CD1c, and CD1d, and process antigens more efficiently than CD11c^lo^ macrophages [97].

Decidual macrophages contribute to the “embryo-friendly” immunological environment, which negates surveillance by immunity and permit embryogenesis, in a similar manner to tumor microenvironment that favors neoplastic growth. Based on their cytokine pattern, they are categorized as M1 and M2, respectively [98, 99]. M1 macrophages secrete tumor necrosis factor-α (TNF-α) and IL-12 [100], while M2 macrophages are characterized by a decreased IL-12 production and express IL-1 receptor antagonist [101]. Additionally, M2 macrophages express the macrophage mannose receptor (MMR) that mediates host defense and plays a role in removal of inflammatory by-products [102]. Human decidual macrophages have been shown to inhibit T-cell responses via prostaglandin E2 production [103, 104]. Furthermore, they produce a significant amount of immunosuppressant IL-10 [105, 106], which can reduce the abortion rate in CBA/J x DBA/2J model, and tryptophan metabolites [107–109], which can effectively promote T_reg_ generation. Taken all the above together, it can be speculated that an M2 phenotype is anticipated during normally developing pregnancy. However, there has been no evidence showing that the decidual macrophages have an immunosuppressive phenotype with M2-polarization.

### Dendritic cells

Dendritic cells (DCs) constitute an essential player which links innate immunity to adaptive immunity. Following antigen capture at the periphery, they migrate to the regional lymph nodes, where they present peptides to naïve T cells, resulting in antigen-specific immune responses [110–113]. Within adaptive immunity, DCs also control polarization of T helper cell differentiation by cytokine secretion; IL-12 from lymphoid DCs induce development of Th1 cells, whereas no convincing factor has been found to induce Th2 development, and polarization to Th17 or Th22 seems not induced only by DCs [114–116]. DCs as antigen-presenting cells are not only essential for the induction of primary immune response but also play a role in the induction of tolerance. Subtypes of DCs in human decidua are described as immature non-activated (CD209^+^), immature activated (DEC205^+^) and mature activated (CD83^+^) cells [117, 118]. Immature DCs, processing antigens via DEC205 receptor, promote CD8^+^ T-cell proliferation but suppress cytotoxic IFN-γ production, resulting in tolerance, whereas mature DCs induce T-cell immunity [117].

There are very few CD83^+^ DCs at the maternal-fetal interface [118], but DCs in pregnant decidua are found CD209^+^, suggesting that recruited monocyte-derived immature DCs are kept non-activated. Furthermore, DCs present in the mouse decidua are unable to migrate out of this tissue even upon activation [119] (Fig. 1). Secreted factors in the conditioned medium of murine decidual cell culture have been shown to block in vivo antigen presentation by DCs and to inhibit their capacity to induce IFN-γ, but not IL-10 production by primed lymphocytes, suggesting that decidual factors contribute to the development of Th2 dominance, through modulation of DCs function [6]. Myeloid DCs in the decidua produce lower levels of IL-12 than their peripheral blood counterparts do and are somewhat more prone to stimulating Th2 responses in human [120]. These results therefore suggest that decidual DCs might locally present antigen to decidual T cells in ways that minimize Th1 responses.

Recent studies suggest that decidual DCs might play a role in decidual tissue remodeling, e.g., DC-deficient mice showed altered decidual angiogenesis [121, 122]. The data on the role of decidual DCs in human pregnancy pathologies are scarce. Only mild changes in decidual CD83^+^ DC densities have been described in human pregnancy complications [123, 124]. Askelund et al. reported on significantly higher number of dendritic cells in deciduas from women with RM at 8-week gestation compared to gestational age-matched normal controls [124].

### Regulatory T cells

CD4^+^CD25^hi^FoxP3^+^ [125] T_reg_ cells are a component of adaptive immunity, and they function as suppressors of the immune response. By their capacity to downregulate immunological reactions, T_reg_ cells are involved in maintenance of self-tolerance, tumor escape, and transplant tolerance, while during pregnancy, under a certain condition, T_reg_ cells can also contribute to maternal tolerance of the fetus via producing IL-10 [126, 127]. Aluvihare et al. suggested for the first time that T_reg_ cells might mediate maternal tolerance in mice during pregnancy [128]. Indeed, adoptive transfer of T_reg_ cells from BALB/c-mated normal pregnant CBA/J mice prevented fetal loss in the abortion-prone DBA/2J-mated CBA/J female mice, while T_reg_ cells from non-pregnant mice had no effect [129]. T_reg_ cells participate in protection of the fetus by down-regulating inflammatory responses. T_reg_ cells inhibit cytokine production in both CD4^+^ T cells and CD8^+^ T cells, cytotoxic activity of NK cells, and dendritic function and maturation, resulting in suppression of local inflammatory activation [127, 130, 131].

The lack of T_reg_ cell-mediated modulation might result in pregnancy failure, or pathologies, but careful distinguish between thymus-derived natural T_reg_ (nT_reg_) cells and induced T_reg_ (iT_reg_) cells generated from peripheral CD4^+^ T cells should be concerned. Reduced frequency of decidual T_reg_ cells was reported in miscarriage with a normal embryo karyotype [132]. In this study, Helios^+^ T_reg_ cells were assessed within CD4^+^FoxP3^+^ population; however, the definition of nT_reg_ cells as Helios^+^CD4^+^FoxP3^+^ is not satisfyingly in consensus. In contrast, Helios^−^ iT_reg_-deficient murine pregnancies are characterized by an increased resorption rate and defective remodeling of spiral arteries [133]. However, in this mouse model, the total abortion rate was only 10 %; thus, indispensability of iT_reg_ cells is not clear. It should also to be noted that there is no evidence that the lack of T_reg_ cells can cause abortion of embryos expressing paternal alloantigens, the depletion of T_reg_ cells in transgenic mice expressing an artificial MHC II-restricted antigen could invoke fetal resorption at mid-pregnancy.

## Non-classical immune communication

Current immunology has revealed intercellular communication not only among lymphoid or myeloid cells but also involving tissue stromal parenchymal or non-parenchymal cells, which can modify the proportion and extracellular paracrine signaling of lymphoid/myeloid cells by means vulnerable to tissue microenvironment. Hereafter, we call this aspect as “non-classical” communication between the embryo and the decidua.

### Roles for the uterine stroma and the decidual cells

**Fig. 3:**
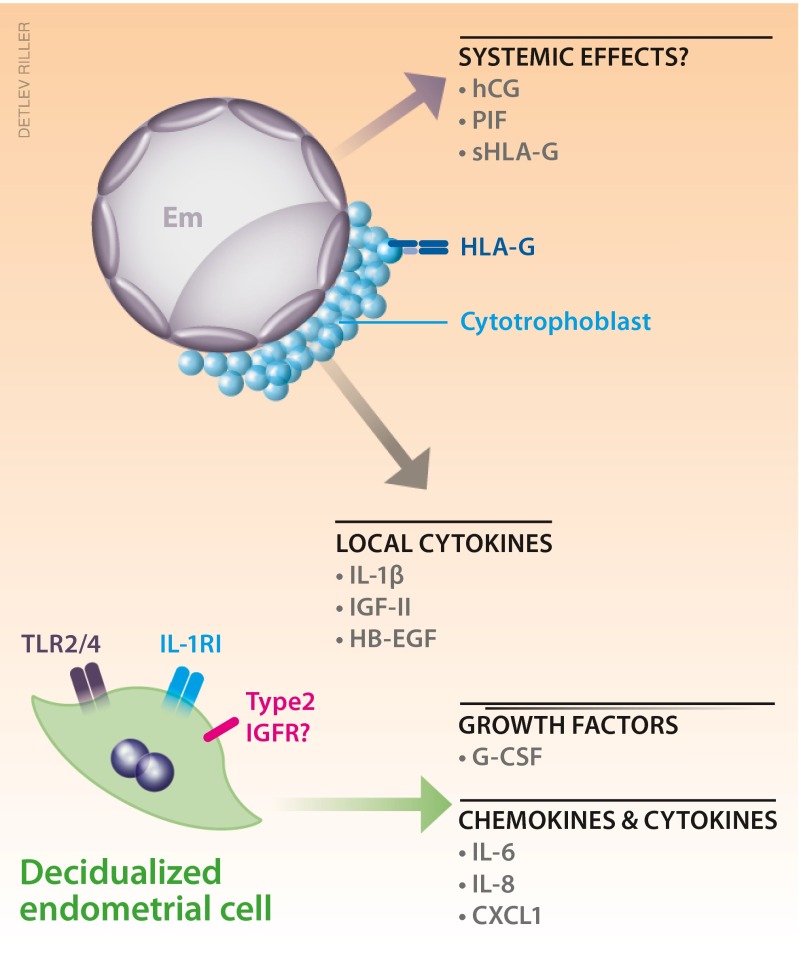
Possible human decidua-embryo interaction indicated by in vitro observation. Despite of difficulty in obtaining human specimen during normal peri-implantation stage, a number of studies have utilized in vitro co-culture system to investigate signaling communication between the decidual cells and the embryo. Fertilized eggs secrete interleukin-1β (IL-1β) and growth factors such as IGF-II and HB-EGF, which are indicated to regulate decidual cellular development, in line with expression of IL-1RI and type 2 IGFR in the endometrial cells. Signaling for decidual expression of Toll-like receptors (TLRs) is unknown. Soluble factors from the embryo but detected in maternal peripheral blood, such as human chorionic gonadotropin (hCG), preimplantation factor (PIF), and soluble form of HLA-G (sHLA-G), may have systemic effects such as promoting ovarian progesterone production, balancing cytokines and chemokines secretion in the periphery. Decidual cells also express growth factors such as G-CSF, and cytokines and chemokines such as IL-6, IL-8, and CXCL1. At least some of them are considered to be under the control of signaling from the embryos

1. Secreted factors from the decidua
  The decidual cells themselves can also play important roles in modulation of immune cells functions. In addition to the diversity of growth factors as decidual markers, cultured endometrial cells secrete cytokines, e.g., IL-6, TNF-α, as well as chemokines, e.g., IL-8, CXCL1, and express the chemokine receptor CXCR4 during normal pregnancy [35, 36]. Miscarriage is characterized by an altered cytokine profile in the human decidua [134]. Uterine stromal fibroblasts produce chemokines, including Cxcl9, Cxcl10, and Ccl5, the production of which is epigenetically downregulated during decidual differentiation upon implantation, by methylation of the promoter regions of the chemokine genes without deactivation of NF-kB or Stat1 signaling, [8]. Therefore, effector cytotoxic CD8^+^ T cells are not permitted to infiltrate into the decidual region adjacent to the conceptus, even if memory T cells against embryonic antigen were experimentally primed on the day after implantation (Fig. 1). Such an epigenetic suppression of chemokines does not take place in the myometrial fibroblasts and stromal cells in non-implantation sites, which suggests the involvement of blastocyst-derived factors. A mouse model of MHC-restricted rejection of fetus accompanied by T cell infiltration is induced by IDO inhibitor 1-methyltryptophan [135]. However, this compound, working as a competitor with the substrate of IDO, tryptophan, may have a diversity of adverse effects, which still raises questions about alternative activation of immunity overcoming the decidual suppression of chemokines suggested by Erlebacher’s group [8]. Apart from cytokines, the dynamic changes in expression levels and patterns of extracellular matrix proteins during the tissue remodeling in early pregnancy [35, 36] might also induce changes in stromal affinity for uNK cells, as suggested by the tissue distribution of uNK cells (Fig. 1).
2. TLR expression on the decidual cells
  There are ten types of human Toll-like receptors (TLRs) with a diversity of specific pattern recognition for pathogens. TLRs are expressed not only in immune cells but also in tissue parenchymal cells such as adipocytes, hepatocytes, and uterine stromal cells. Changes of uterine TLR expression during the menstrual cycle may suggest hormonal regulation [136] (Fig. 3). Although only a few studies have shown the existence of TLR2 and TLR4 in human decidual cells [137], Krikun et al. have reported that LPS stimulation induces secretion of IL-6 and IL-8 in human endometrial cell culture [138]. It cannot be ruled out that if bacterial infection stimulates TLR2/4 on decidual cells during pregnancy and downstream signaling induces cytokine production, endogenous lipid ligands for TLR2/4 present on uterine stromal cells modify the character of these cells during decidualization. The mechanisms for the regulation of cytokine secretion by stromal or decidual cells requires further investigation.

### Refrained contact from the embryo to maternal immune cells

Importantly, dying or incompetent embryo such as that with abnormal karyotype, seen in humans more frequently than in mice, will be “rejected” by the decidua, the development of which requires calcium signaling via serine proteases secreted from a competent embryo. When endometrial cells are exposed to culture supernatant of incompetent embryos, impaired secretion of prolactin and IGFBP-1 is caused via endoplasmic reticulum (ER) stress and autophagy [139]. The cytotrophoblasts of human embryos at peri-implantation stage also secrete IL-1β (Fig. 3). In baboons, IL-1β has been shown to promote decidual secretion of matrix metalloprotease [140]. Growth factors such as insulin-like growth factor II (IGF-II) and heparin-binding epidermal growth factor (HB-EGF) are indicated to regulate decidual cellular development, in line with expression of IL-1RI and type 2 IGFR in the endometrial cells [140, 141]. Extravillous trophoblast cells, which invade deep into the decidua, express non-canonical class I HLA-E and HLA-G (Fig. 3) in addition to canonical HLAs, which increase Foxp3 expression of T_reg_ cells and generation of CD45RA^+^ resting T_reg_ cells [142]. However, this does not necessary mean that these HLAs are indispensable to establish uterine receptivity against embryos. Soluble factors from the embryo but detected in maternal peripheral blood, such as human chorionic gonadotropin (hCG), preimplantation factor (PIF), and soluble form of HLA-G (sHLA-G), may have systemic effects such as promoting ovarian progesterone production, balancing cytokine and chemokine secretion in the periphery (Fig. 3) [143–146]. A genomics study revealed that PIF, produced by the embryo post-fertilization, upregulates CX3CL1 expression in the cultured decidual cells from first-trimester pregnant women, and increases interleukin-1 receptor-associated kinase 1-binding protein 1 (IRAK1BP1) expression in both decidual culture and human endometrial stromal cells (hESCs) [147]. However, the gene or enzyme responsible for PIF production is unknown, sustaining studies on PIF KO mice unavailable, which will be necessary to be challenged in order to understand the importance of this peptide.

## Conclusions: toward clinical endpoints

### Current diagnosis for infertility and failures in treatment

Defective decidua formation in early pregnancy may result in infertility or in a later onset of complications such as preeclampsia, recurrent abortion, and pre-term birth [18, 148, 149]. The definition of infertility by the World Health Organization (WHO) is childlessness within 1 year of active sexual intercourse [150]. Risk factors for female infertility and subfertility including recurrent abortion are uterine disorders such as endometriosis (10 % incidence) [151] and uterine fibroids (70∼80 % women affected, but only a small proportion with huge lesions suffer infertility due to physical unreceptivity) [152], or ovarian problems, e.g., polycystic ovarian syndrome (PCOS) in 4∼8 % women in reproductive age [153] and premature ovarian failure in 0.8∼3.7 % women of various races between 40 and 45 years old [154]. In some cases, infertility is treatable such as surgical resection of fibroids, or metformin administration for PCOS patients with insulin tolerance to reduce androgen’s suppressive effect on luteal development. However, there are still 42 % of RM still unaccounted for (excluding 41 % abnormal embryonic karyotype and recalculated from original literature [155]). According to Japanese governmental reports, the number of successful cases in assisted reproduction technologies (ART) doubled between 2002 and 2012 (15,228 cases in 2002 and 37,953 cases in 2012) [156], while the number of total infertile patients increased four times during the same period (85,664 women in 2002 and 326,426 women in 2012) [156].

Increased number of ART can be attributed to the progress and advances in in vitro fertilization (IVF), intracytoplasmic sperm injection (ICSI), and utilization of frozen eggs. Another effort from immunological view has been made using intravenous immunoglobulin (IVIg) administration to the patients with autoimmune disease represented by anti-phospholipid (aPL) syndrome, systemic lupus erythematosus (SLE), autoimmune thyroid disease (AITD), and type 1 diabetes mellitus [157]. However, this approach has been recognized as the final choice for subgroups of infertile women who were not rescued by other means, because of the high risk of adverse effects and the swelling medical costs [157]. Furthermore, indeed aPL antibodies have affinity to the surface phospholipid exposed on cytotrophoblasts, and indeed IVIg treatment can rescue the RM in AITD patients but in combination with thyroid hormones [157], the mechanisms how autoantibodies affect implantation and how IVIg rescues are unclear. In most cases in autoimmune diseases, ovarian functions and other endocrinal system are also affected. Typical remedy with combination of IVIg and NSAIDs may just suppress cytokine storms, but without understanding the basis it is hard to select appropriate patients.

### Better understanding the mechanisms via optimal animal models

A number of molecules, e.g., leukemia inhibitory factor (LIF) and lipid mediator prostaglandins, associated with “uterine receptivity” have been identified in PR and ER pathways and Wnt signaling, via gene-deficient murine models [18]. Immunologists in the field of reproduction have utilized T, B, NK-deficient Rag2^−/−^Δγc or T, NK-deficient tgε26 to elucidate the role of immune cells in decidual vascular remodeling failure. However, these immunodeficient mice did not show complete pregnancy loss, with only modest changes in uterine vascular remodeling or decidual cellularity, let alone the high resorption rate and placental shrinkage in tgε26 mice [158], probably due to the compensatory functions of other minor immune cellular populations and of uterine stromal cells, or to the adaptively enhanced vascular development, and also to the absence of cytotoxic immune cells in the maternal side. Moreover, only in later placental stage of pregnancy do these mice show increased resorption and abnormal placenta, with an essential caveat of fetal genotype’s effects. Thus, wild-type embryo transfer to these uteri will better clarify the maternal cells roles. In contrast, decidual defects have been reported in several gene-targeted mouse models (Hoxa10, Hoxa11, Bmp2, Wnt4, Dedd, and IL11ra) [13–16, 19, 23]. Several mouse models deficient in cytokine signaling such as Lif^−/−^ or Stat3^−/−^ show infertile phenotype [159, 160], but the defects are already found in implantation rates before decidualization, suggesting more fundamental and pleiotropic roles for the signaling downstream. Intriguingly, uterine conditional Trp53^−/−^ mice present decidual senescence accompanied with excessive terminal differentiation and multi-nuclearization until later gestational period, which results in preterm birth [161]. In order to shed more light on intrinsic and mutual roles for maternal immune cells, uterine vascular and stromal cells, it is necessary to generate new combination of different mouse models. A mouse model with uterine decidual deficiency named as above in combination with NK-deficient models may more clearly explain the functions of uNK cells on decidual development and vice versa, via crossing the two mouse lines and via transferring wild-type NK cells. Likewise, uterine vascular deficiency models might shed light on the role for vascular factors in uNK cell functions. However, embryonic or postnatal lethality of Vegfa^−/−^, Angpt1^−/−^, and Angpt2^−/−^ mice demands to assess uterine conditional deletion of these genes, which has not been challenged for female reproductive functions. For instance, PR-Cre x Vegfa^flox^ will delete decidual cell-specific deletion of VEGF-A, and VE-cadherin-Cre-ER x Vegfa^flox^ is possible to delete vascular endothelial VEGF-A production via tamoxifen administration.

Rigorous studies on basic and clinical sides are required to overcome conflicting evidences (which are partially due to the lack of human samples at the peri-implantation stage), in order to obtain a molecular clue for the causes of infertility, and to invent novel diagnostic methods and treatments.
